# A two-year progress report on medical education standards in the Western Pacific: Presidential address 2018

**DOI:** 10.3352/jeehp.2018.15.6

**Published:** 2018-03-09

**Authors:** Michael Field

**Affiliations:** Emeritus Professor, Sydney Medical School, University of Sydney, Sydney, NSW, Australia; Hallym University, Korea

It’s with pleasure that I provide an update on the activities and plans of our organisation since my last report in the *Journal of Educational Evaluation for Health Professions* in February 2016 [1]. In doing so, I need first to mention that a process of re-branding was undertaken in 2017, to simplify our association’s name, and to develop a new logo and website. The name chosen was the Western Pacific Association for Medical Education (WPAME). A fresher logo and clearer website design were introduced in conjunction with this name change, as can be seen at our website http://www.wpame.org.au.

The aim of the Association remains unchanged, namely to provide coordination and leadership towards the goal of enhancing the quality of medical education in the countries of the Western Pacific region (as defined by World Health Organization [WHO]). This role is consistent with our status as one of the 6 regional associations of the World Federation for Medical Education (WFME).

## Contributions to regional conferences

The WPAME meets at least once each year to review its activities and progress its agenda, and it does so in conjunction with established conferences in the field of medical education being held in the region, to allow members to gain benefit from participation in both events.

**Fig. 1.**
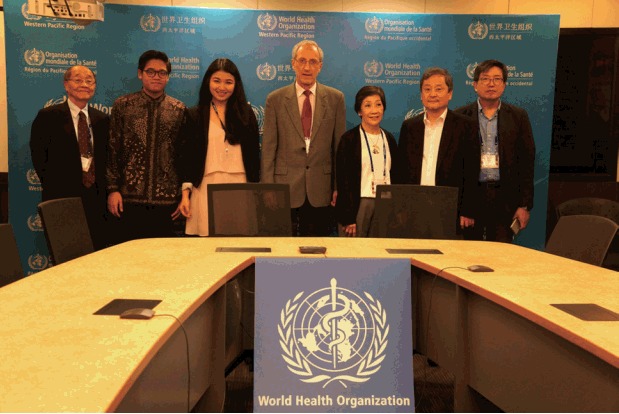
The author (centre) with members of the Association for Medical Education in the Western Pacific Region (Western Pacific Association for Medical Education) delegation visiting the Western Pacific Regional Office of World Health Organization, Manila, February 2017.

In February 2017, the Association was invited to participate in the 50 years anniversary convention of the Association of Philippine Medical Colleges, held in Manila. In addition to contributing 2 plenary lectures to the main conference program, we also presented a Symposium addressing the issue of medical ethics and professionalism education in countries of the Western Pacific Region. The opportunity was also taken to tour the headquarters of the Western Pacific Regional Office of WHO, located in Manila, and this was beneficial in increasing understanding and cooperation between our two organisations in the area of health workforce development (Fig. 1).

Our meeting in 2018 was conducted at the Asia Pacific Medical Education Conference held in Singapore in January. Attending the many sessions of this excellent conference was of great benefit to our members, and we were also pleased to present a symposium entitled “Required resources for learning: a Western Pacific regional perspective.” A well-attended meeting of our Advisory Board was also conducted during the course of this conference.

It’s also worth recording that both I as President and our past President Ducksun Ahn were invited to attend the 2016 Conference of the International Association of Medical Regulatory Authorities in Melbourne, where we addressed a Policy Roundtable on Health Workforce Regulation and Education conducted by the WHO Western Pacific Regional Office. This gave our organisation the opportunity to develop further the important relationship between medical school accreditation and the regulation of medical practitioners.

## Medical program evaluations

In July 2017, a WPAME team conducted a progress evaluation visit to the National University of Samoa in Apia, supported by the WHO Western Pacific Regional Office. This was a progress assessment following the consultative visit to the new Faculty of Medicine at the University which we had conducted in September 2015. The interactions during the visit and the report provided were greatly valued by the leadership of the Faculty and the University, as well as by representatives of the Government, and are likely to strongly influence the further development of this new school.

## National consultations

In December 2017, WPAME was invited to send a speaker to the first Vietnam National Medical Education Conference, held in Ho Chi Minh City. The Secretary-Treasurer of the Association Pete Ellis attended, and his presentation on accreditation had a significant impact on the thinking of academic and political leaders who were present. Follow-up of this contribution will occur through the WPAME Board member for Vietnam, professor Tuan Tran, who is participating in a Reform Committee which is developing plans for quality assurance of medical education through the Vietnam Ministry of Health.

A number of preliminary requests for support and advice have been received from other countries in the region, and these will be followed up as required.

## World Federation for Medical Education links

As a regional association of the WFME, the WPAME participates in global initiatives of the Federation, and sends its President to the annual meetings of the WFME Executive Council. I was pleased to attend and contribute to these meetings in Melbourne in September 2016 and in Geneva in May 2017.

## Next phase of leadership

The terms of the current officeholders of WPAME come to an end on 30 June 2018, and the individuals forming the new leadership team were decided at the recent meeting of the Advisory Board. The incoming President will be Dujeepa Samarasekera of Singapore, and the Vice President will be Pete Ellis of New Zealand. I will be pleased to take the role of immediate past President, and am confident that the Association will continue in strong hands in the years ahead.
